# Supplementary material for: Apparent bias toward long gene misregulation in MeCP2 syndromes disappears after controlling for baseline variations
Source: Nat Commun. 2018 Aug 13;9:3225. doi: 10.1038/s41467-018-05627-1 (PMC6089998; doi:10.1038/s41467-018-05627-1)
Supplement: Supplementary file 1 — Supplementary Information [file 41467_2018_5627_MOESM1_ESM.pdf]

Supplementary Information

Title: Apparent bias towards long gene misregulation in MeCP2 syndromes disappears after controlling for baseline variations

Authors: Raman et al

Graduate Program in Quantitative and Computational Biosciences, Baylor College of Medicine, Houston, TX 77030, USA; Jan and Dan Duncan Neurological Research Institute at Texas Children's Hospital, Houston, Texas 77030, USA

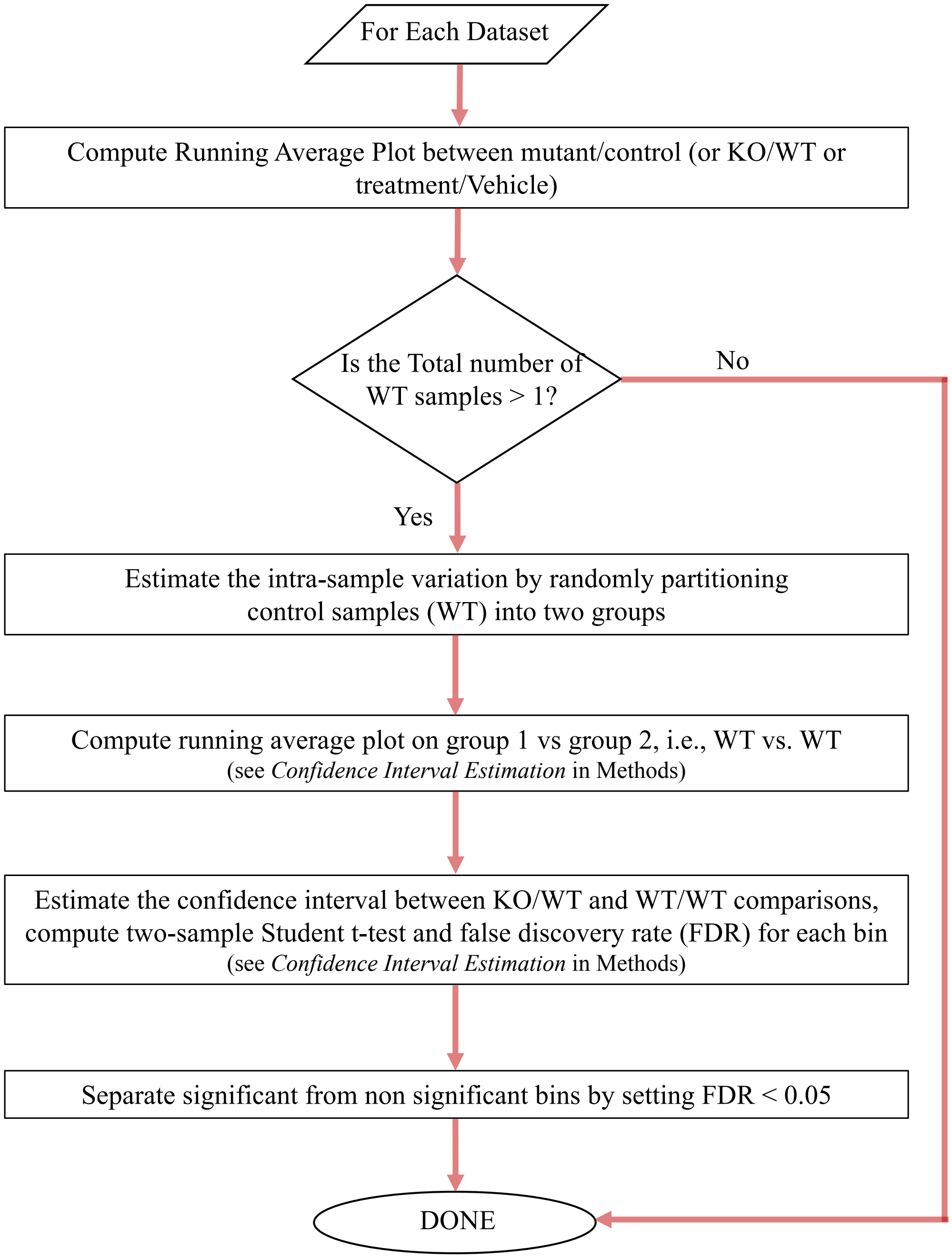

Schematic for rigorous assessment of long gene trends

Supplementary Figure 1. A schematic diagram of the proposed work-flow for rigorous assessment of long gene trends

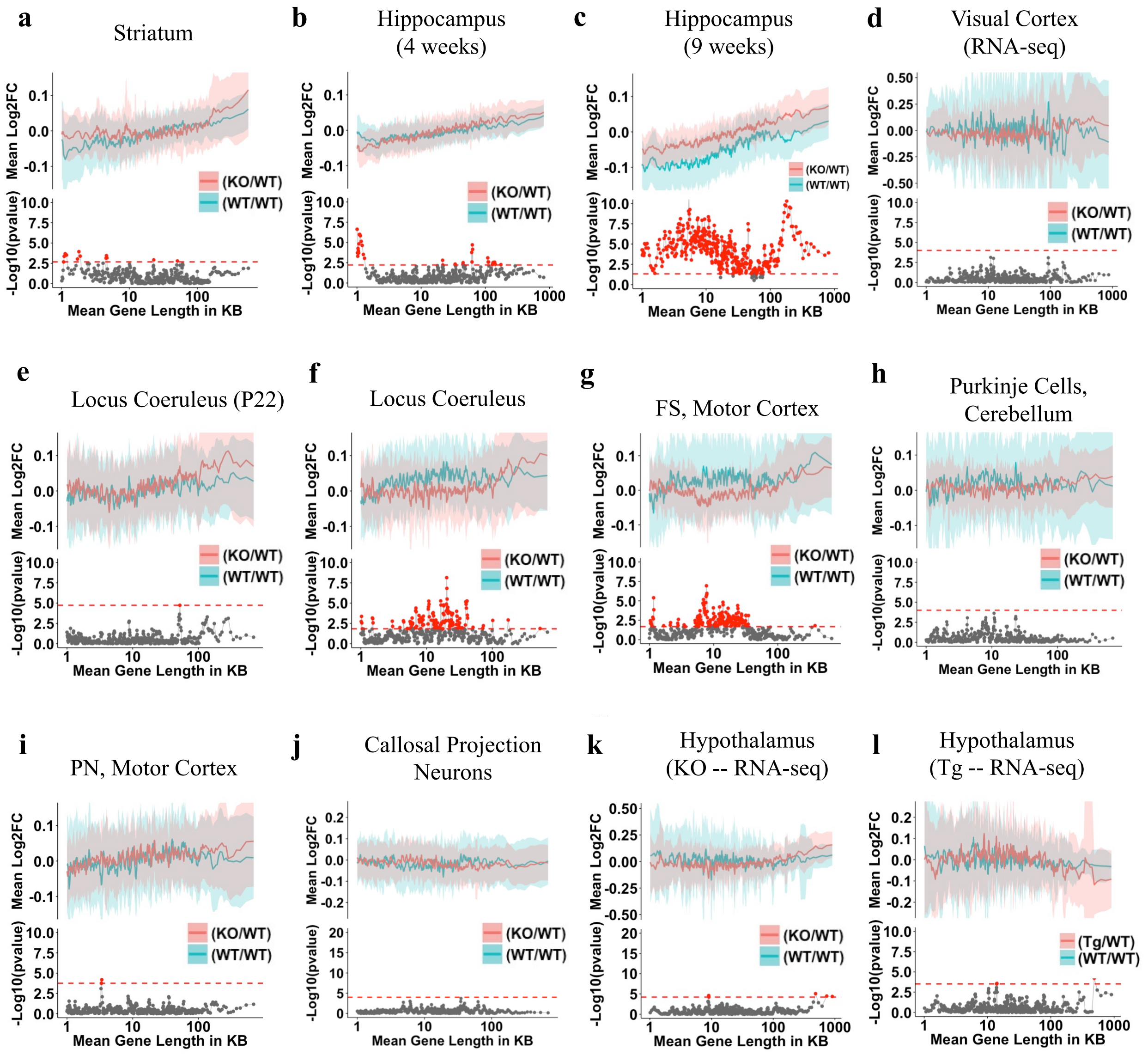

Supplementary Figure 2. Consistent and significant long gene trends are not present in MeCP2 datasets. (**a-l**) Analysis of intra-sample variation in WT *Mecp2* dataset shows a bias toward long genes across different brain regions. The blue lines (BL) show the comparison of permuted WT/WT samples for each respective dataset (please see the comparison table below for sample details). The red line (RL) represents the comparison of KO or MUT or Tg samples to WT littermates from a respective dataset (as described in the comparison table below). The top half of each subgraph shows the lines that represent fold-change in expression for genes binned according to gene length (bin size of 200 genes with shift size of 40 genes) as previously described[1]. Note that we observe few long gene bins as well as short gene bins with significant preferential upregulation in *Mecp2*-null mice datasets. The blue and red ribbon correspond to one-half of one standard deviation of each bin for the comparison of WT/WT (blue) and

KO/WT or Tg/WT (red ribbons). The bottom half of each subgraph is the p-value from the two-sample t-test between KO/WT or Tg/WT and WT/WT. Bins with FDR < 0.05 are shown in red. The red dotted line indicates the minimum -Log_{10}(p-value) that corresponds to a FDR < 0.05. List of comparisons for Supplementary Figure 2 are present in Supplementary Table 1 below:

Brain region	Mouse lines compared	Reference
a. Striatum	BL: C57BL WT vs C57BL WT (n = 2 each)<br>RL: C57BL KO vs C57BL WT (n = 5 each)	Zhao et al. *Neuro of Disease* 2013[2]
b. Hippocampus (4 weeks)	BL: FVBx129 WT vs FVBx129 WT (n = 2 each)<br>RL: FVBx129 KO vs FVBx129 WT (n = 4 each)	Baker et al. *Cell* 2013[3]
c. Hippocampus (9 weeks)	BL: FVBx129 WT vs FVBx129 WT (n = 2 each)<br>RL: FVBx129 KO vs FVBx129 WT (n = 4 each)	Baker et al. *Cell* 2013[3]
d. Visual Cortex	BL: WT vs WT (n = 1 each)<br>RL: KO (Mecp2tm1.1Bird) vs WT (n = 3 each)	Gabel, Kinde et al. *Nature* 2015[1]
e. Locus Coeruleus Neurons (TH Young/~P22)	BL: C57BL/6J WT vs C57BL/6J WT (n = 1 each)<br>RL: C57BL/6J KO vs C57BL/6J WT (n = 3 each)	Sugino et al. *J Neurosci.* 2014[4]
f. Locus Coeruleus Neurons (TH)	BL: C57BL/6J WT vs C57BL/6J WT (n = 1 each)<br>RL: C57BL/6J KO vs C57BL/6J WT (n = 3 each)	Sugino et al. *J Neurosci.* 2014[4]
g. Fast Spiking interneurons, Motor Cortex (G42)	BL: C57BL/6J WT vs C57BL/6J WT (n = 2 each)<br>RL: C57BL/6J KO vs C57BL/6J WT (n = 4 each)	Sugino et al. *J Neurosci.* 2014[4]
h. Purkinje Cells, Cerebellum (G42)	BL: C57BL/6J WT vs C57BL/6J WT (n = 1 each)<br>RL: C57BL/6J KO vs C57BL/6J WT (n = 3 each)	Sugino et al. *J Neurosci.* 2014[4]
i. Pyramidal Neurons, Motor Cortex (YPFH)	BL: C57BL/6J WT vs C57BL/6J WT (n = 1 each)<br>RL: C57BL/6J KO vs C57BL/6J WT (n = 3 each)	Sugino et al. *J Neurosci.* 2014[4]
j. Callosal Projection Neurons	BL: C57BL/6J WT vs C57BL/6J WT (n = 1 each)<br>RL: C57BL/6J KO vs C57BL/6J WT (n = 3 each)	Kishi et al. *Nature Comm.* 2016[5]
k. Hypothalamus (KO – RNA-seq)	BL: FVBx129SvEvTac WT vs FVBx129SvEvTac WT (n =1 each)<br>RL: FVBx129SvEvTac KO vs FVBx129SvEvTac WT (n = 3 each)	Chen et al. *PNAS* 2015[6]
l. Hypothalamus (Tg – RNA-seq)	BL: FVBx129SvEvTac WT vs FVBx129SvEvTac WT (n =1 each)<br>RL: FVBx129SvEvTac Tg vs FVBx129SvEvTac WT (n = 3 each)	Chen et al. *PNAS* 2015[6]

Supplementary Table 1: List of comparisons used in the overlap plots in Supplementary Figure 2. BL and RL stands for Blue line and Red line respectively.

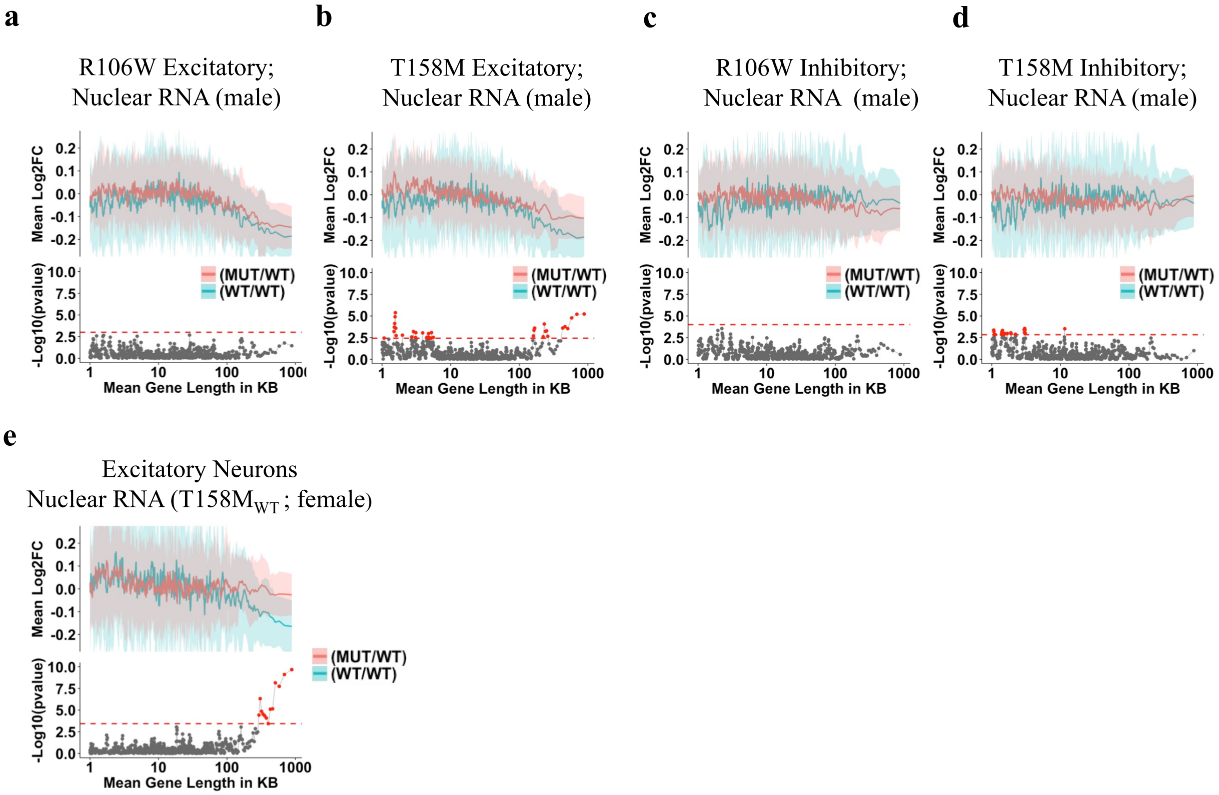

Supplementary Figure 3. Long-gene trends are not observed in different *Mecp2* mutation types, neuronal subtypes, or genders. (**a-e**) Blue lines (BL) represent the comparison of permuted WT/WT samples from each dataset (as mentioned in the comparison table). The red lines (RL) represent the comparison of MUT samples to WT littermates for the respective dataset (as mentioned in the comparison table). The top half of each subgraph shows the lines that represent fold-change in expression for genes binned according to gene length (bin size of 200 genes with shift size of 40 genes) as described[1]. The blue and red ribbons correspond to one-half of one standard deviation of each bin for the comparison of WT/WT and MUT/WT respectively. The bottom half of each subgraph is the p-value from the two-sample t-test between MUT/WT and WT/WT. Bins with FDR < 0.05 are shown in red. The red dotted line indicates the minimum -Log_{10}(p-value) that corresponds to a FDR < 0.05. List of comparisons for Supplementary Figure 3 are present in Supplementary Table 2 below:

Brain region	Mouse lines compared	Reference
a. Cortical Excitatory Neurons (R106W, Male)	BL: C57BL/6J WT vs C57BL/6J WT (n = 2 each)<br>RL: C57BL/6J MUT vs C57BL/6J WT (n = 4 each)	Johnson et al. *Nature Med.* 2017[7]
b. Cortical Excitatory Neurons (T158M, Male)	BL: C57BL/6J WT vs C57BL/6J WT (n = 2 each)<br>RL: C57BL/6J MUT vs C57BL/6J WT (n = 4 each)	Johnson et al. *Nature Med.* 2017[7]
c. Cortical Inhibitory Neurons (R106W, Male)	BL: C57BL/6J WT vs C57BL/6J WT (n = 2 each)<br>RL: C57BL/6J MUT vs C57BL/6J WT (n = 4 each)	Johnson et al. *Nature Med.* 2017[7]
d. Cortical Inhibitory Neurons (T158M, Male)	BL: C57BL/6J WT vs C57BL/6J WT (n = 2 each)<br>RL: C57BL/6J MUT vs C57BL/6J WT (n = 4 each)	Johnson et al. *Nature Med.* 2017[7]
e. Excitatory Neurons ($T158M_{WT}$; Female)	BL: C57BL/6J WT vs C57BL/6J WT (n = 1 each)<br>RL: C57BL/6J MUT vs C57BL/6J WT (n = 2 each)	Johnson et al. *Nature Med.* 2017[7]

Supplementary Table 2: List of comparisons used in the overlap plots in Supplementary Figure 3. BL and RL stand for Blue line and Red line respectively.

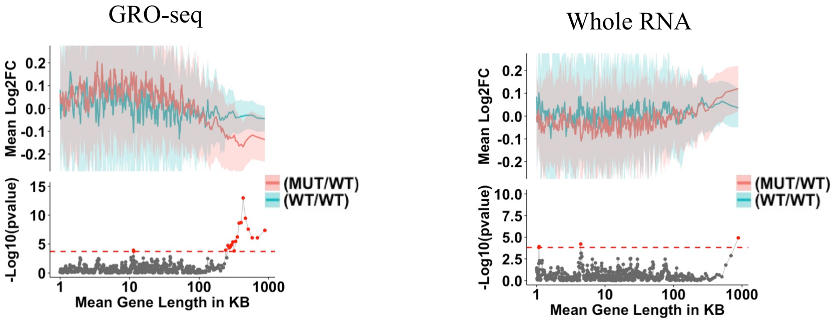

Supplementary Figure 4. Intra-sample variation in WT samples from *Mecp2* datasets is independent of the RNA isolation method. Blue lines (BL) represent the comparison of permuted WT/WT samples from each dataset (please see the comparison table below for details). The red lines (RL) represent the comparison of MUT samples to WT littermates from each dataset (see comparison table below). The top half of each subgraph shows the lines that represent fold-change in expression for genes binned according to gene length (bin size of 200 genes with shift size of 40 genes) as previously described[1]. The blue and red ribbons correspond to one-half of one standard deviation of each bin for the comparison of WT/WT and MUT/WT, respectively. The bottom half of each subgraph is the p-value from the two-sample t-test between MUT/WT and WT/WT. Bins with FDR < 0.05 are shown in red. The red dotted line indicates the minimum -Log$_{10}$(p-value) that corresponds to a FDR < 0.05. List of comparisons for Supplementary Figure 4 are present in Supplementary Table 3 below:

Brain region	Mouse lines compared	Reference
Male Cortex (GRO-Seq)	BL: C57BL/6J WT vs C57BL/6J WT (n = 1 each)<br>RL: C57BL/6J R106W vs C57BL/6J WT (n = 2 each)	Johnson et al. *Nature Med.* 2017[7]
Male Cortex (Whole Cell)	BL: C57BL/6J WT vs C57BL/6J WT (n = 1 each)<br>RL: C57BL/6J R106Wvs C57BL/6J WT (n = 2 each)	Johnson et al. *Nature Med.* 2017[7]

Supplementary Table 3: List of comparisons used in the overlap plots in Supplementary Figure 4. BL and RL stand for Blue line and Red line respectively.

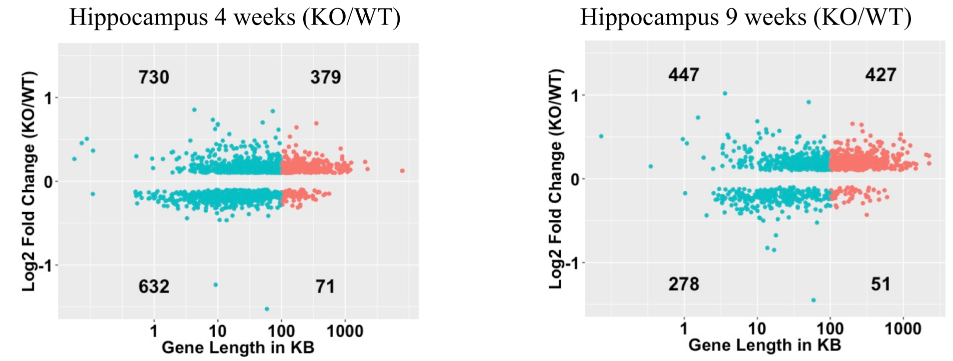

Supplementary Figure 5. Gene expression data from *Mecp2*-mutant hippocampi. Differentially expressed gene analysis using the data from Baker et al., 2013 derived from the hippocampi of *Mecp2*-null male mice. Scatter plot of log fold-change (log2FC > 0.1 and FDR < 0.05) in expression between FVBx129 KO to its FVBx129 WT littermates (y-axis) against its gene length (x-axis) in samples of hippocampus from 4-week old and 9-week old mice (n = 4 each)[3].

a **SEQC RNA-seq**

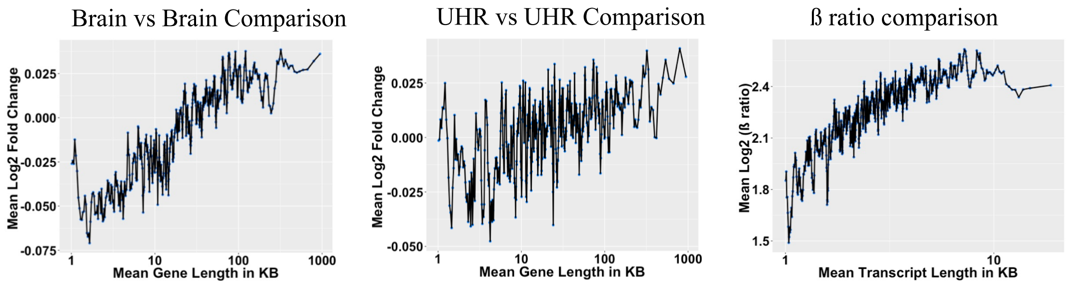

b **SEQC Array**

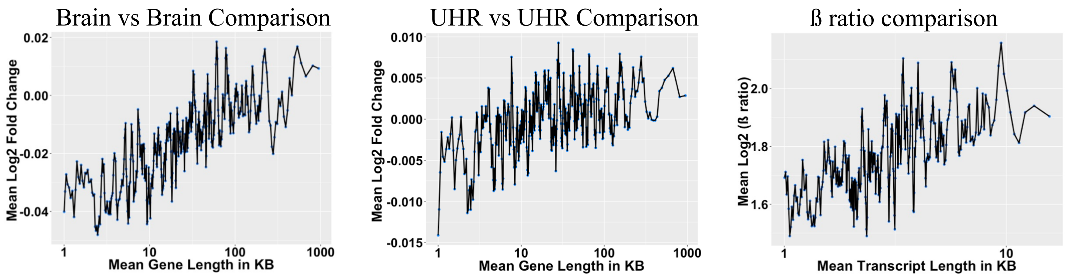

Supplementary Figure 6. A long gene bias is detected in the SEQC datasets. (**a**) Novartis SEQC[8] RNA-seq data. Left panel: Brain vs. Brain randomized $\log_2$ fold-change in gene expression plotted against gene length (n = 32 each). Middle panel: Universal Human Reference (UHR) vs UHR randomized log fold-change in gene expression plotted against gene length (n = 32 each). Right panel: $\log_2$ β ratio plotted against transcript length using the SEQC RNA-seq dataset (right panel; n = 32 each). (**b**) SEQC microarray data. Left panel: Brain vs. Brain randomized fold-change plotted against gene length (n = 2 each). Middle panel: Universal Human Reference (UHR) vs UHR randomized log fold-change plotted against gene length (n=2 each). Right panel: $\log_2$ β ratio plotted against transcript length using the SEQC microarray dataset (n = 2 each). Each blue dot is a bin of 200 genes with shift size of 40 genes, consistent with previous analyses[1].

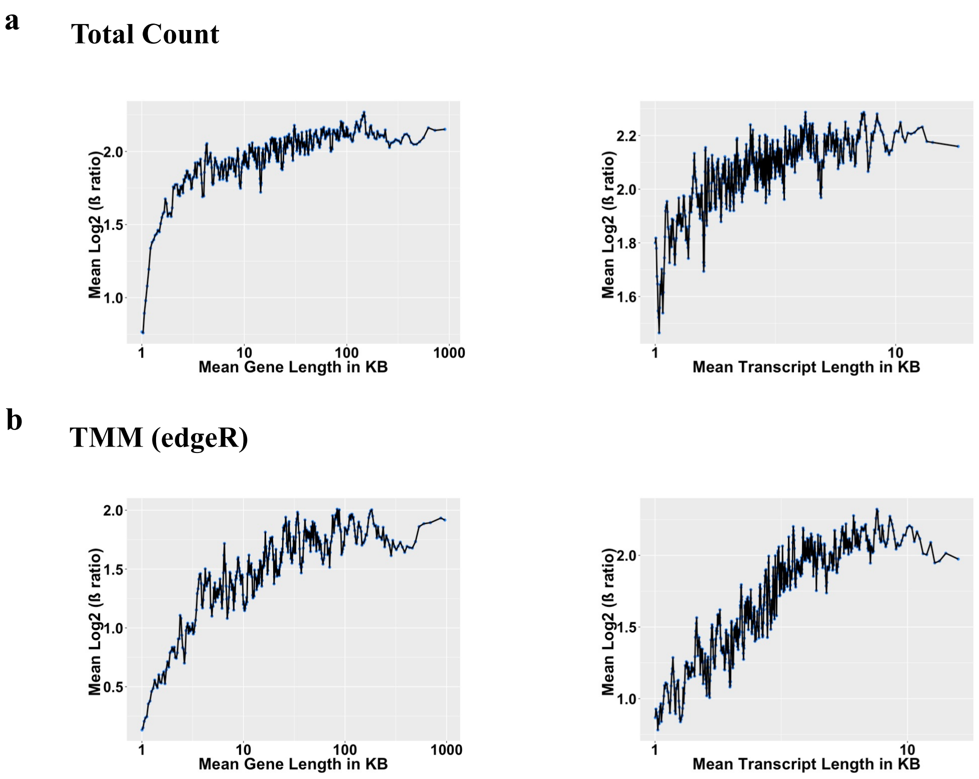

Supplementary Figure 7. Long gene bias is independent of normalization methods. Data is from the SEQC RNA-seq samples (n = 64 each). The Log2 β ratio was calculated after utilizing different normalization methods and plotted against gene length or transcript length. (**a**) Mean log2 β ratio after library size normalization (or total count) plotted against gene length (left panel) & transcript length (right panel). (**b**) Mean log2 β ratio after TMM[9] (edgeR[10]) normalization plotted against gene length (left panel) & transcript length (right panel). Each blue dot is a bin of 200 genes with shift size of 40 genes, consistent with previous analyses[1]**.**

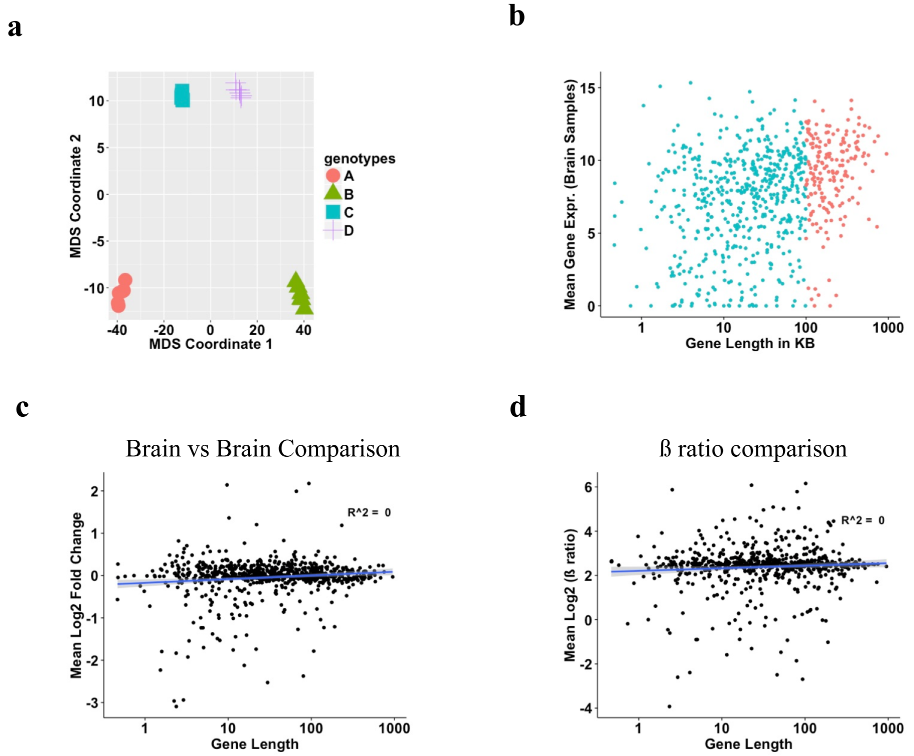

Supplementary Figure 8. A long gene bias is not observed in the Nanostring dataset. (**a**) PCA plot of the NanoString dataset (n = 6 for each sample type). (**b**) Scatter plot of mean gene expression in brain samples plotted against gene length. (**c**) brain vs. brain randomized fold-change in gene expression plotted against gene length (n = 6 each). (**d**) Mean log2 β ratio plotted against gene length, where the β ratio = (B-A/C-A) = 4:1 (n = 6 each for each sample type).

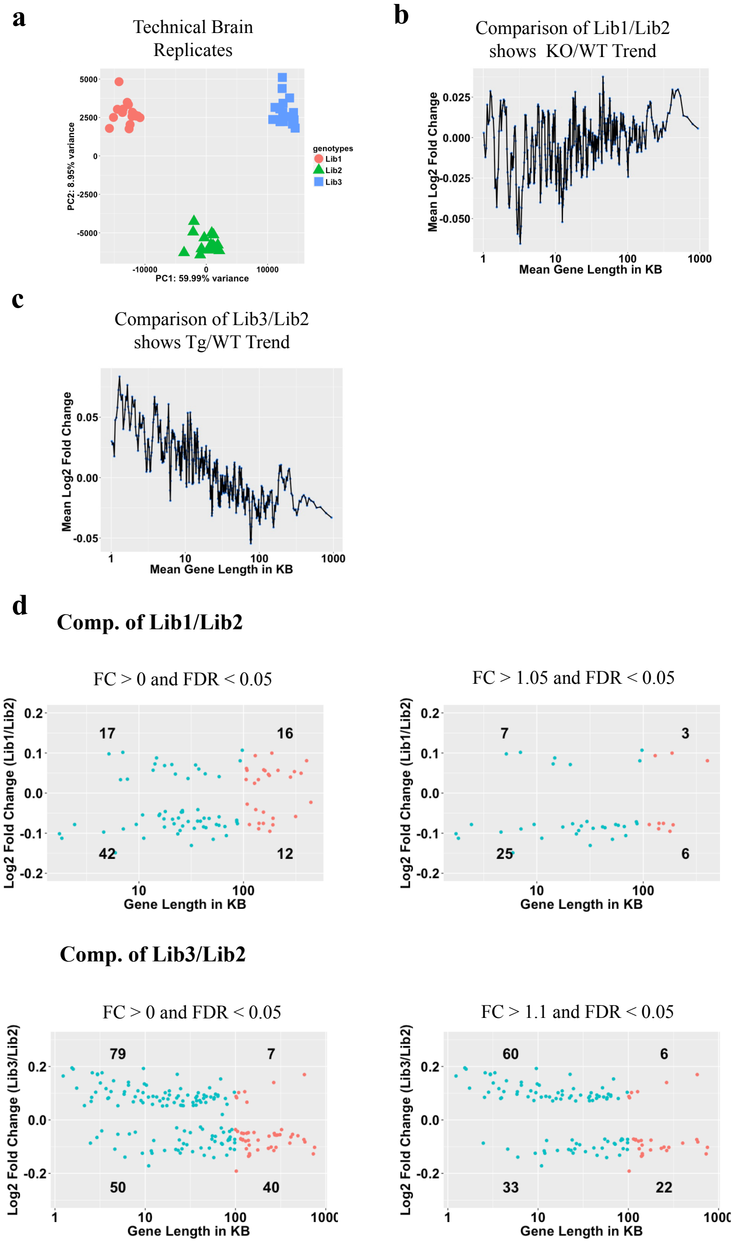

Supplementary Figure 9. A possible explanation of reciprocal relationship among transcriptional changes between RTT and *MECP2* duplication syndrome. (**a**) PCA Plot of the B samples in Novartis SEQC RNA-seq dataset using library prep IDs. (**b**) Comparison of gene expression levels in brain samples from library preparation 1 vs. library preparation 2 plotted against gene length (n = 16 each). Each blue dot is a bin of 200 genes with shift size of 40 genes, consistent with previous analyses[1]. (**c**) Comparison of gene expression levels in brain samples from library preparation 3 vs. library preparation 2 plotted against gene length (n = 16 each). Each blue dot is a bin of 200 genes with shift size of 40 genes,

consistent with previous analyses[1]. (**d**) Differential expression analysis (using DESeq2) between brain samples having library preparation 1 vs. library preparation 2 (upper two panels) and library preparation 3 vs. 2 (bottom two panels) across different fold changes and with an FDR < 0.05 (using 750 genes present in the Nanostring PanCancer Human Dataset). The red and blue dots indicate long and short genes, respectively.

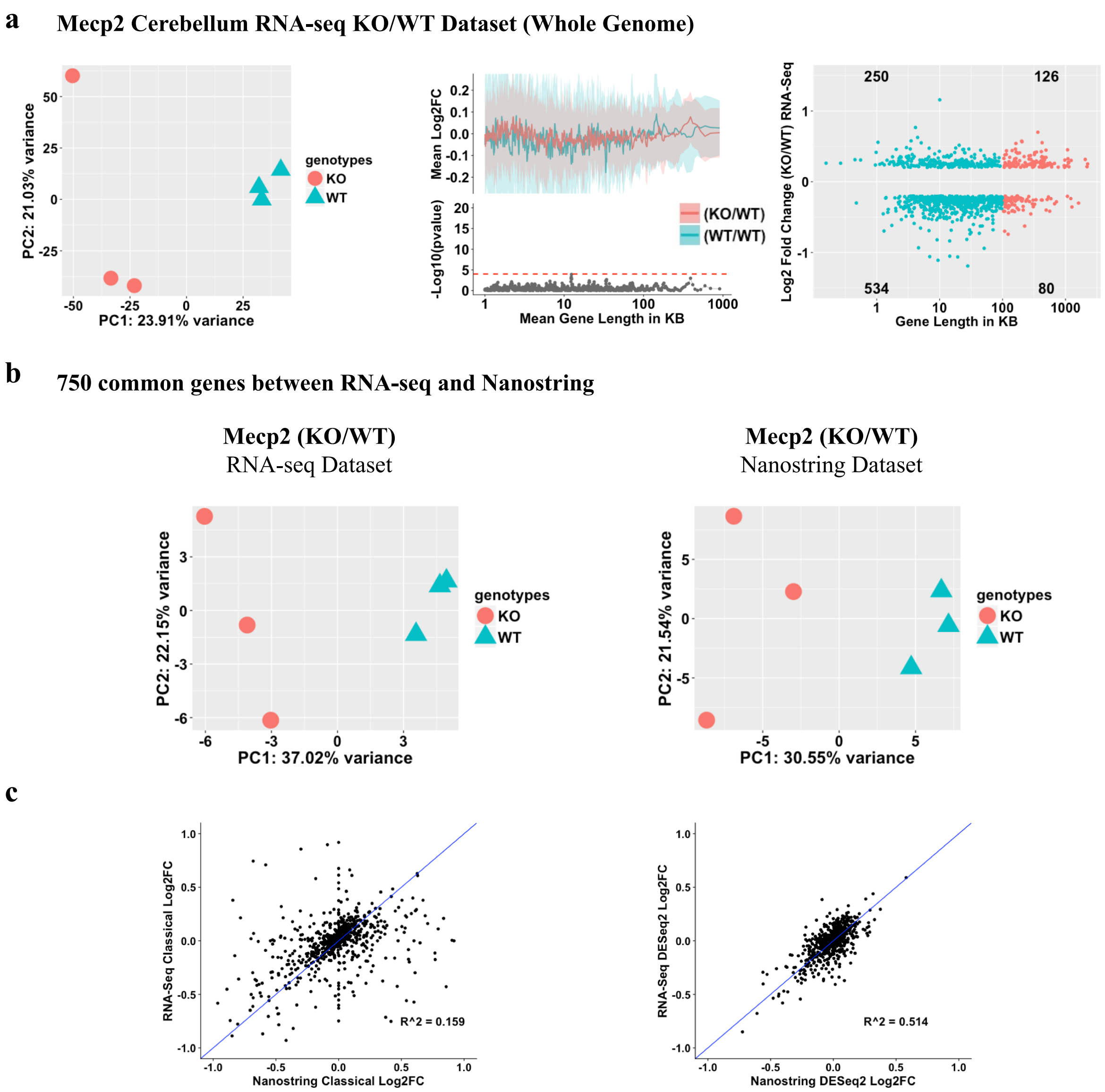

Supplementary Figure 10. RNA-seq and Nanostring analysis of *Mecp2*-KO and WT male cerebellum samples. (**a**) Left panel: PCA analysis using all the genes in the RNA-seq cerebellum dataset. PCA Plot of *Mecp2*-KO and WT samples (n = 3 per genotype). Middle panel: running average and overlap plots. The blue line (BL) represents the comparison of permuted WT/WT samples from the cerebellum dataset (n = 1 each). The red line (RL) represents the comparison of KO samples to WT littermates (n = 3 each). The top half of each subgraph shows the lines that represent fold-change in expression for genes binned according to gene length (bin size of 200 genes with shift size of 40 genes) as described[1]. The blue and red ribbon correspond to one-half of one standard deviation of each bin for the comparison of WT/WT and KO/WT respectively. The bottom half of each subgraph is the p-value from

the two-sample t-test between KO/WT and WT/WT. Bins with FDR < 0.05 are shown in red. The red dotted line indicates the minimum $-Log_{10}$(p-value) that corresponds to a FDR < 0.05. Right panel: scatter plot of log fold-change in expression between KO and WT samples (n = 3 each) plotted against gene length. Only differentially expressed genes (FDR < 0.05 & absolute log2FC > log2(1.2)) were plotted. (**b**) Analysis using the 750 genes quantified in both the RNA-seq and NanoString datasets. PCA plot of *Mecp2*-KO and WT samples (n = 3 each) for the RNA-seq (left panel) and NanoString (right panel) platforms. (**c**) Comparison of log2 fold-changes using the classical/standard method (left panel) and the shrunken log_2 fold-changes method (right panel).

Supplementary References

1 Gabel, H. W. *et al.* Disruption of DNA-methylation-dependent long gene repression in Rett syndrome. *Nature* 522, 89-93 (2015).

2 Zhao, Y.-T., Goffin, D., Johnson, B. S. & Zhou, Z. Loss of MeCP2 function is associated with distinct gene expression changes in the striatum. *Neurobiol Dis* 59, 257-266 (2013).

3 Baker, S. A. *et al.* An AT-hook domain in MeCP2 determines the clinical course of Rett syndrome and related disorders. *Cell* 152, 984-996 (2013).

4 Sugino, K. *et al.* Cell-Type-Specific Repression by Methyl-CpG-Binding Protein 2 Is Biased toward Long Genes. *J Neurosci* 34, 12877-12883 (2014).

5 Kishi, N. *et al.* Reduction of aberrant NF-κB signalling ameliorates Rett syndrome phenotypes in Mecp2-null mice. *Nat Commun* 7, 10520 (2016).

6 Chen, L. *et al.* MeCP2 binds to non-CG methylated DNA as neurons mature, influencing transcription and the timing of onset for Rett syndrome. *Proc Natl Acad Sci USA* 112, 5509-5514 (2015).

7 Johnson, B. S. *et al.* Biotin tagging of MeCP2 in mice reveals contextual insights into the Rett syndrome transcriptome. *Nat Med* 23, 1203-1214 (2017).

8 SEQC/MAQC-III, C. A comprehensive assessment of RNA-seq accuracy, reproducibility and information content by the Sequencing Quality Control Consortium. *Nat Biotechnol* 32, 903-914 (2014).

9 Robinson, M. D. & Oshlack, A. A scaling normalization method for differential expression analysis of RNA-seq data. *Genome Biol* 11, R25 (2010).

10 Robinson, M. D., McCarthy, D. J. & Smyth, G. K. edgeR: a Bioconductor package for differential expression analysis of digital gene expression data. *Bioinformatics* 26, 139-140 (2010).
