# Supplementary material for: Apparent bias toward long gene misregulation in MeCP2 syndromes disappears after controlling for baseline variations
Source: Nat Commun. 2018 Aug 13;9:3225. doi: 10.1038/s41467-018-05627-1 (PMC6089998; doi:10.1038/s41467-018-05627-1)
Supplement: Supplementary file 3 — Description of Additional Supplementary Files [file 41467_2018_5627_MOESM3_ESM.pdf]

Description of Additional Supplementary Files

File Name: Supplementary Data 1
Description: Detailed description of all the datasets used in this paper.

File Name: Supplementary Data 2
Description: Description of RNA-seq iPSC and RTT dataset. The samples were generated by Dr. Bill Lowry's lab at UCLA.

File Name: Supplementary Data 3
Description: Description of RNA-seq dataset of *Mecp2*-KO and WT male cerebellum samples. The samples were generated by Dr. Huda Zoghbi's lab at BCM.

File Name: Supplementary Data 4
Description: Description of NanoString dataset of *Mecp2*-KO and WT male cerebellum samples. The samples were generated by Dr. Huda Zoghbi's lab at BCM.
